# Assessment of Knowledge and Practice Gaps in the Prevention of Contrast-Associated Acute Kidney Injury Among Doctors of Multiple Specialties in a Tertiary Care Hospital: A Clinical Audit

**DOI:** 10.7759/cureus.115141

**Published:** 2026-08-25

**Authors:** Sritharan Thivacaren, Anura P Hewageegana

**Affiliations:** 1 Department of Nephrology, National Hospital of Sri Lanka, Colombo, LKA

**Keywords:** contrast-associated acute kidney injury, intravenous hydration, knowledge scores, practice gaps, radiology

## Abstract

Introduction: Contrast-associated acute kidney injury (CA-AKI) is a common and potentially preventable cause of hospital-acquired acute kidney injury. International guidelines recommend risk assessment, appropriate hydration, medication review, and patient counseling to reduce its incidence. However, adherence to these recommendations in routine clinical practice remains uncertain.

Methods: A criterion-based clinical audit was conducted among 102 doctors from multiple specialties at the National Hospital of Sri Lanka. An anonymous online questionnaire assessed knowledge and self-reported practices against Kidney Disease Improving Global Outcomes (KDIGO) and European Society of Urogenital Radiology recommendations. Descriptive statistics evaluated compliance with predefined audit standards, while Kruskal-Wallis and Spearman rank correlation analyses explored differences among professional groups and the relationship between knowledge and clinical practice.

Results: The mean knowledge score was 3.61/4, with registrars achieving the highest mean score (3.79/4). Most participants correctly identified the KDIGO definition of CA-AKI (89.2%), the diagnostic timeframe (90.2%), and intravenous hydration as the primary preventive strategy (91.2%). However, important practice deficiencies were identified in medication review (angiotensin-converting enzyme inhibitor withholding, 50.0%), appropriate hydration strategies for patients with heart failure (44.1%), and routine patient counseling (61.8%). No significant correlation was observed between knowledge scores and counseling practice (Spearman's ρ = 0.004, p = 0.97).

Conclusion: Although clinicians demonstrated good theoretical knowledge of CA-AKI prevention, substantial gaps in evidence-based clinical practice were identified. These findings support the implementation of standardized institutional protocols, targeted education, and repeat audit cycles to improve guideline adherence and enhance patient safety.

## Introduction

Contrast-associated acute kidney injury (CA-AKI) represents a significant challenge in modern clinical medicine. As the use of diagnostic and interventional radiology increases globally, iodinated contrast media have become a cornerstone of patient management. CA-AKI is characterized by a sudden decline in renal function following the intravascular administration of iodinated contrast, and it remains the third most common cause of hospital-acquired acute kidney injury [1].

The clinical implications of CA-AKI are profound; affected patients face extended hospital stays, increased healthcare costs, a heightened risk of developing chronic kidney disease (CKD), and increased mortality [2]. Data from Sri Lanka suggest that CA-AKI continues to occur at a clinically significant rate in tertiary care settings, particularly among patients with preexisting renal impairment, diabetes mellitus, and those undergoing arterial contrast procedures [3]. Despite these risks, many cases of CA-AKI are preventable through guideline-based care. This clinical audit assessed healthcare professionals’ knowledge against Kidney Disease Improving Global Outcomes (KDIGO) [4] and European Society of Urogenital Radiology (ESUR) recommendations to identify practice gaps and improve patient safety.

## Materials and methods

This criterion-based clinical audit was conducted at the National Hospital of Sri Lanka from February to March 2026. Audit standards were derived from KDIGO Clinical Practice Guidelines for Acute Kidney Injury [4] and the ESUR Guidelines. The key audit standards evaluated include the following: 1) mandatory preprocedural risk assessment for CA-AKI, 2) recommendation of adequate intravenous hydration for at-risk patients, 3) temporary discontinuation or review of nephrotoxic medications prior to contrast exposure, and 4) identification of procedural risks (contrast volume, osmolarity, and repeated exposure). The target benchmark compliance was set at ≥80% correct responses for each key knowledge and practice domain.

A total of 102 doctors participated in the audit. The sample included Intern Medical Officers, Residential House Officers, Medical Officers, Registrars, and Senior Registrars. Participants were drawn via a convenience approach from diverse clinical specialties involved in ordering contrast investigations, including Nephrology, Cardiology, Critical Care, Emergency Medicine, and General Medicine. Data were collected using an anonymous, self-administered online questionnaire hosted on Google Forms (Google LLC, Mountain View, CA). To ensure content validation and address the lack of standard prevalidated metrics, the survey instrument was developed following a comprehensive review of the KDIGO and ESUR guidelines [4,5]. The draft questionnaire was subjected to an expert panel review by senior clinicians in nephrology and radiology to assess its clarity, clinical relevance, and alignment with audit standards. Pilot testing was conducted among a small cohort of nonparticipating medical professionals (n = 5) to refine readability and ensure format validity prior to formal deployment. The full questionnaire is provided in the Appendix. Ethical approval was obtained from the National Hospital Sri Lanka, and informed consent was acquired from all participants. The questionnaire comprised four domains:

1) Demographic profile: Designation, specialty, years of experience, frequency of contrast requests.

2) Basic knowledge: Assessing the ability to identify the KDIGO definition of CA-AKI [4] and the typical onset window (24-72 hours).

3) Risk and prevention: Evaluating knowledge of the strongest predictors (preexisting CKD), contrast-related risk factors, and the most effective preventive strategies (intravenous hydration).

4) Clinical practice: Measuring self-reported adherence to preprocedure estimated glomerular filtration rate (eGFR) assessment, patient counseling, and hydration protocols for specific patient cohorts (e.g., heart failure).

Knowledge score was derived from four predefined knowledge questions about KDIGO definition, timing of CA-AKI, risk factors, and preventive Strategies. Questions about eGFR assessment prior to contrast administration, patient counseling, and hydration protocols for high-risk patients evaluated clinicians' self-reported practice outcomes.

Data analysis

Data were exported to Microsoft Excel (Microsoft Corporation, Redmond, WA) and analyzed using descriptive statistics (percentages and mean scores) to preserve maximum clinical granularity, item-by-item, against the ≥80% benchmark. Total knowledge scores (ranging from 0 to 4) were used as secondary, broad indices. Data were nonnormally distributed (Shapiro-Wilk test, p < 0.001); therefore, the Kruskal-Wallis and Spearman rank correlation analyses were performed using Python 3.11 (SciPy and Stats models; Python Software Foundation, Wilmington, DE).

## Results

**Figure 1:**
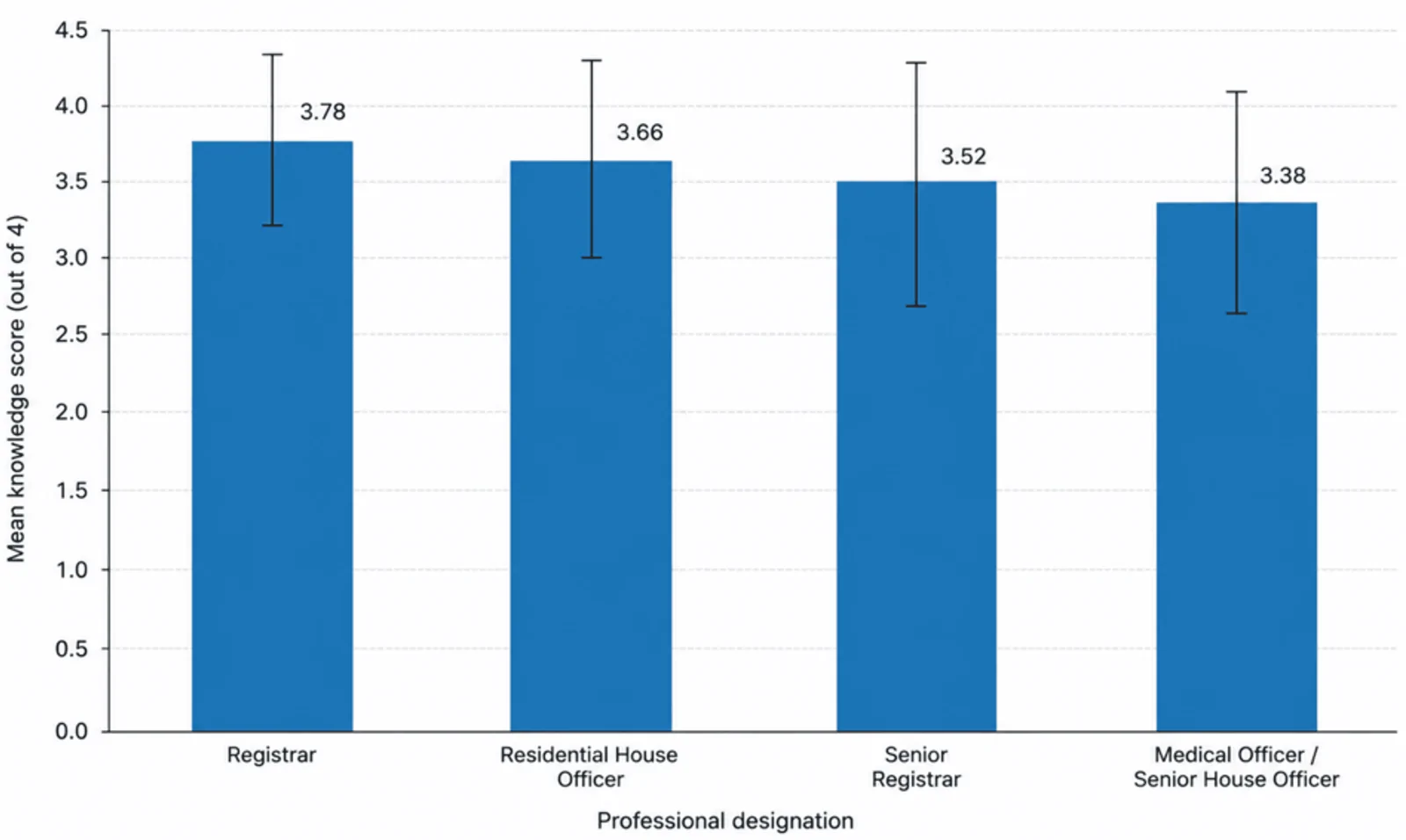
Mean knowledge scores according to professional designation. Data are presented as means with 95% confidence intervals and vertical error bars

**Figure 2:**
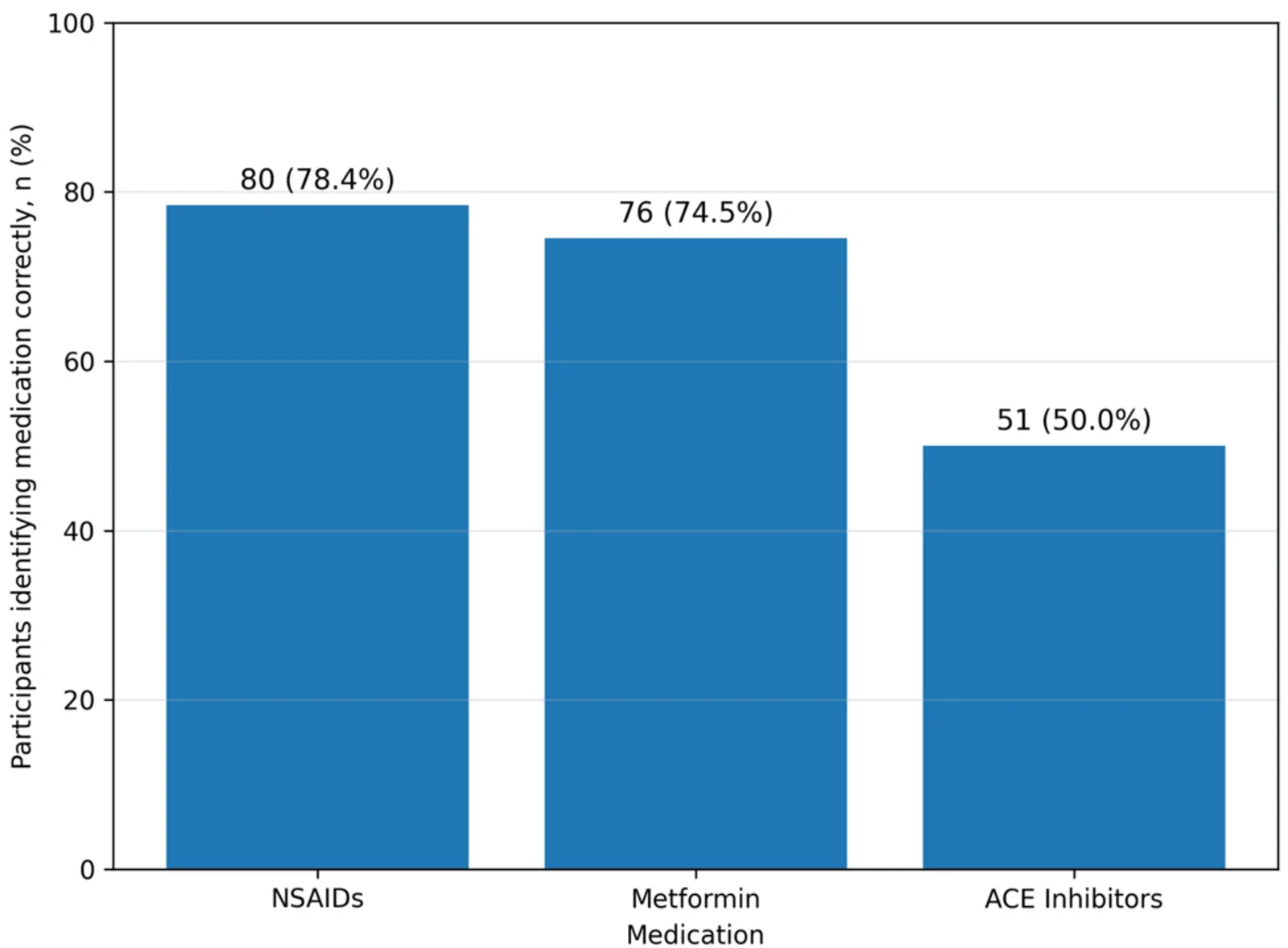
Participants' knowledge regarding medications requiring withholding before contrast administration. Data are presented as n (%) NSAIDs: nonsteroidal anti-inflammatory drugs; ACE: angiotensin-converting enzyme

**Figure 3:**
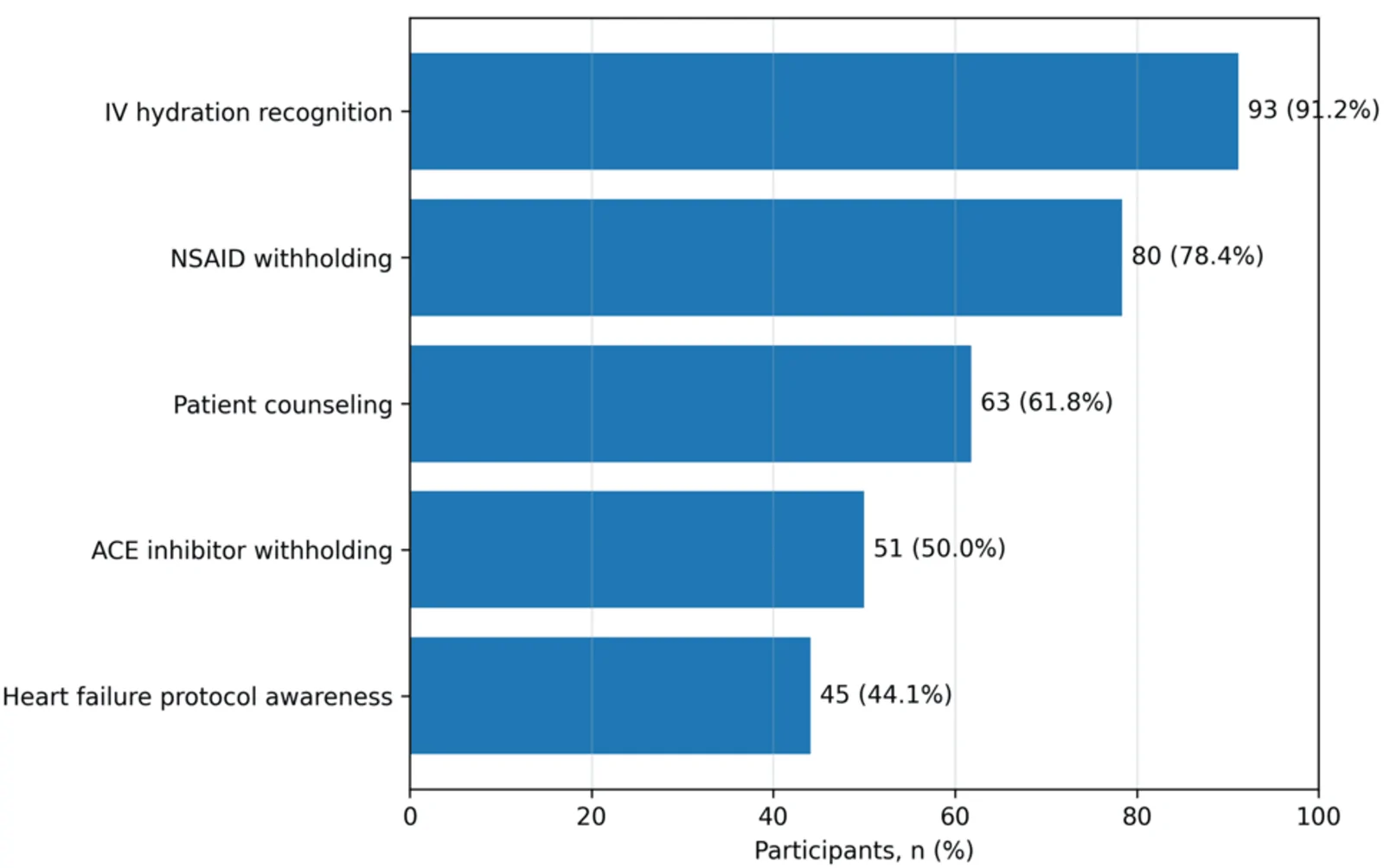
Key gaps in knowledge and clinical practice related to CA-AKI prevention, highlighting discrepancies between awareness and implementation. Data are presented as n (%) CA-AKI: contrast-associated acute kidney injury; IV: intravenous; NSAID: nonsteroidal anti-inflammatory drug; ACE: angiotensin-converting enzyme

A total of 102 doctors completed the evaluation. The mean knowledge score across the entire cohort was 3.61 out of 4 (Figure 1). Registrars achieved the highest descriptive mean score (3.79/4). Knowledge of basic concepts of CA-AKI, including the KDIGO definition and diagnostic time frame, was demonstrated by 91 (89.2%) and 92 (90.2%) of participants, respectively. Intravenous isotonic fluid administration was recognized as the most effective preventive intervention by 93 participants (91.2%). Important deficiencies were identified across key practice domains (Table 1). While 80 (78.4%) participants correctly identified nonsteroidal anti-inflammatory drugs that require temporary discontinuation, only 51 (50.0%) recognized the need to temporarily discontinue angiotensin-converting enzyme (ACE) inhibitors before contrast administration, representing a major 30% gap below the audit standard (Figure 2). Routine preprocedural patient counseling was reported by 63 (61.8%), and appropriate hydration strategies for patients with heart failure were identified by only 45 (44.1%), which was critically low (Figure 3). Although preexisting renal impairment and hypovolemia were widely recognized as risk factors, 94 (92.2%), repeated contrast exposure within 72 hours was identified by only 89 (87.3%). However, specific procedural risks related to contrast volume and osmolarity fell far short of the target audit criteria, with minimal recognition among nonradiology staff.

**Table 1:** Compliance with key audit criteria for the prevention of CA-AKI (n = 102) Data are presented as n (%) CA-AKI: contrast-associated acute kidney injury; KDIGO: Kidney Disease Improving Global Outcomes; IV: intravenous; NSAIDs: nonsteroidal anti-inflammatory drugs; ACE: angiotensin-converting enzyme

Audit criterion	Participants meeting the criterion, n (%)
Correct identification of the KDIGO definition of CA-AKI	91 (89.2)
Correct identification of the diagnostic window (24-72 hour)	92 (90.2)
Recognition of IV hydration as the primary preventive strategy	93 (91.2)
Correct identification of NSAIDs requiring withholding before contrast administration	80 (78.4)
Recognition of the need for patient counseling	63 (61.8)
Correct identification of ACE inhibitors requiring withholding before contrast administration	51 (50.0)
Correct identification of the appropriate hydration protocol in patients with heart failure	45 (44.1)

Knowledge scores, assessed using nonparametric analysis, showed no significant association with professional seniority or years of clinical experience, suggesting that these deficiencies were widespread across all grades. Spearman rank correlation showed no significant correlation between good theoretical knowledge and self-reported patient counseling practices (ρ = 0.004, p = 0.97), indicating that higher knowledge did not translate into better clinical practice.

## Discussion

This clinical audit demonstrated that although doctors had high theoretical knowledge regarding CA-AKI, critical deficiencies existed in translating this knowledge into routine clinical practice. More than 90% of participants correctly identified the KDIGO definition, diagnostic time frame, and intravenous isotonic hydration as the cornerstone of prevention [4]. However, compliance with several key audit standards, including medication review, individualized hydration in heart failure, and routine patient counseling, remained well below the predefined benchmark of 80%. These findings indicate a clinically significant knowledge-practice gap.

The high level of knowledge regarding the diagnosis and prevention of CA-AKI is uplifting and reflects the widespread adoption of international guidelines. Both the KDIGO and ESUR guidelines emphasize risk assessment, identification of high-risk patients, and isotonic intravenous hydration as the most effective preventive strategy [5]. The outstanding performance in these domains suggests that clinicians are familiar with current evidence-based recommendations.

Despite high theoretical knowledge, critical deficiencies were identified in medication management and risk-adapted hydration strategies. Routine discontinuation of ACE inhibitors remains controversial [6]; current guidelines recommend individualized assessment in high-risk patients because these agents may alter renal autoregulation during periods of hemodynamic stress [6]. In our local setting, temporary withholding of ACE inhibitors and metformin is a commonly adopted precautionary practice, particularly given the relatively high prevalence of mild baseline renal impairment and multiple comorbidities among our patient population. Only 44.1% correctly identified appropriate hydration strategies for patients with heart failure. This finding is clinically important, as concern about fluid overload frequently leads to inappropriate prophylactic hydration [7]. Despite routine guideline recommendations favoring a carefully monitored, individualized approach to fluid administration, adherence to appropriate fluid management strategies may be challenging in patients with comorbidities such as heart failure.

A key finding of this audit was the absence of any correlation between theoretical knowledge and self-reported counseling practice (Spearman's ρ = 0.004, p = 0.97). This objectively confirms that the observed knowledge-practice gap is a system-wide issue rather than an individual educational deficit. Implementation research has shown that clinician behavior is influenced by organizational factors such as workload, lack of standardized protocols, time constraints, and absence of clinical reminders, which often limit adherence to evidence-based guidelines [8]. Therefore, system-level interventions, including standardized clinical checklists, regular educational programs, and electronic decision support tools, are needed to improve adherence to international guidelines [9]. A repeat audit will be undertaken to evaluate the effectiveness of these interventions.

This audit has limitations. It was conducted in a single tertiary care center and relied on self-reported practice rather than direct observation, introducing the possibility of reporting bias. Convenience sampling may have introduced selection bias, as clinicians with greater interest in CA-AKI may have been more likely to participate; therefore, the findings may not fully represent the knowledge and practices of all clinicians in the Institution. In addition, patient outcomes such as the incidence of CA-AKI were not assessed. Nevertheless, inclusion of doctors from multiple specialties involved in ordering contrast-enhanced investigations provides a comprehensive assessment of institutional practice.

## Conclusions

Despite high theoretical knowledge, critical deficiencies in medication management and risk-adapted hydration strategies reveal a significant knowledge-practice gap. This gap has important clinical consequences, including risks to patient safety and an increased like hood of preventable acute kidney injury. These findings highlight the need for structured institutional protocols system level interventions and prospective reaudit to improve compliance with international recommendations and enhance patient safety.

## Appendices

**Table 2:** Audit questionnaire. Section 1: sociodemographic data ICU: intensive care unit

Question no.	Question	Response options
Consent	Do you agree to proceed with the questionnaire?	Yes; No
1	Designation	Intern Medical Officer; Residential House Officer; Medical Officer/Senior House Officer; Registrar; Senior Registrar; Other
2	Current practicing specialty (working area)	Cardiology; Nephrology; Radiology; Critical Care (ICU); Emergency Medicine; Surgery; Other
3	Gender	Male; Female
4	Years of experience	<2 years; 2-5 years; 6-10 years; >10 years
5	Approximately how many patients under your care required contrast imaging/procedures during the past month?	None; 1-5 patients; 6-10 patients; 11-20 patients; >20 patients

**Table 3:** Audit questionnaire. Section 2: knowledge about CA-AKI and associated risk factors CA-AKI: contrast-associated acute kidney injury; KDIGO: Kidney Disease Improving Global Outcomes; ACE: angiotensin-converting enzyme; CKD: chronic kidney disease; eGFR: estimated glomerular filtration rate

Question no.	Question	Response options
6	Which of the following best defines CA-AKI according to the KDIGO AKI criteria?	A. Any deterioration in renal function after iodinated contrast; B. Increase in serum creatinine ≥0.3 mg/dL within 48 hours or ≥1.5× baseline within 7 days; C. Transient reduction in urine output immediately after contrast; D. Fall in urine output of any duration after contrast; E. Any increase in serum creatinine within 7 days after contrast
7	Usual time for development of CA-AKI after contrast exposure	Within 6 hours; 24-72 hours 5-7 days; After two weeks; After one month
8	Which single factor most strongly predicts CA-AKI risk?	Obesity; Diabetes mellitus; Sepsis; Pre-existing renal impairment (CKD/AKI); Young age
9	Which of the following are risk factors for CA-AKI? (Select all that apply)	Hypovolemia; Nephrotoxic medications; Advanced age (>70 years); Intra-arterial administration; Diabetes mellitus
10	Which of the following are contrast-related risk factors? (Select all that apply)	High-osmolar contrast; Multiple contrast administrations within 72 hours; Low-osmolar contrast; Intra-arterial administration with renal first pass; Low-volume contrast
11	Most effective strategy to prevent CA-AKI	N-acetylcysteine; High-dose statins; Adequate intravenous hydration (Normal saline), Intravenous bicarbonate; Oral hydration
12	Which medications are usually withheld before contrast administration in high-risk patients? (Select all that apply)	ACE inhibitors; nonsteroidal anti-inflammatory drugs; Beta blockers; Metformin; Diuretics
13	Recommended hydration protocol in high-risk patients (Select all that apply)	0.9% Normal saline 1 mL/kg/h for 6 hours before and after elective contrast; 0.9% Normal saline with rate adjusted for heart failure; 0.9% Normal saline 3 mL/kg/h for 1 hour before emergency contrast; Intravenous bicarbonate 1 mL/kg/h for 6 hours before and after contrast; Intravenous Hartmann solution 1 mL/kg/h for 6 hours before and after contrast
14	Which statements are true regarding CKD (eGFR <30 mL/min) and urgent contrast procedures? (Select all that apply)	Proceed with contrast using an appropriate hydration protocol if urgent; Can only be performed after pre-procedure dialysis; Consider alternative imaging modalities if available; Repeat serum creatinine after 24-72 hours; Urgent dialysis immediately after the procedure without waiting for renal function

**Table 4:** Audit questionnaire. Section 3: practice and attitude eGFR: estimated glomerular filtration rate

Question no.	Question	Response options
15	I routinely assess renal function/document eGFR before requesting contrast imaging	Always; Often; Sometimes; Never; I don't know
16	I counsel patients about the risk of kidney injury before contrast administration	Always; Often; Sometimes; Never; I don't know
